# Hyperlipidemic Conditions Impact Force-Induced Inflammatory Response of Human Periodontal Ligament Fibroblasts Concomitantly Challenged with *P. gingivalis*-LPS

**DOI:** 10.3390/ijms22116069

**Published:** 2021-06-04

**Authors:** Judit Symmank, Sophie Appel, Jana Asisa Bastian, Isabel Knaup, Jana Marciniak, Christoph-Ludwig Hennig, Annika Döding, Ulrike Schulze-Späte, Collin Jacobs, Michael Wolf

**Affiliations:** 1 Department of Orthodontics, University Hospital Jena, Leutragraben 3, 07743 Jena, Germany; sophie.katharina@gmx.de (S.A.); Christoph-Ludwig.Hennig@med.uni-jena.de (C.-L.H.); collin.jacobs@med.uni-jena.de (C.J.); 2 Department of Orthodontics, University Hospital RWTH Aachen, Pauwelsstraße 30, 52074 Aachen, Germany; abastian@ukaachen.de (J.A.B.); iknaup@ukaachen.de (I.K.); jana_marciniak@hotmail.de (J.M.); michwolf@ukaachen.de (M.W.); 3 Section of Geriodontics, Department of Conservative Dentistry and Periodontics, University Hospital Jena, Leutragraben 3, 07743 Jena, Germany; Annika.Doeding@med.uni-jena.de (A.D.); Ulrike.Schulze-Spaete@med.uni-jena.de (U.S.-S.)

**Keywords:** periodontitis, obesity, inflammation, orthodontic tooth movement, periodontal ligament fibroblasts

## Abstract

In obese patients, enhanced serum levels of free fatty acids (FFA), such as palmitate (PA) or oleate (OA), are associated with an increase in systemic inflammatory markers. Bacterial infection during periodontal disease also promotes local and systemic low-grade inflammation. How both conditions concomitantly impact tooth movement is largely unknown. Thus, the aim of this study was to address the changes in cytokine expression and the secretion of human periodontal ligament fibroblasts (HPdLF) due to hyperlipidemic conditions, when additionally stressed by bacterial and mechanical stimuli. To investigate the impact of obesity-related hyperlipidemic FFA levels on HPdLF, cells were treated with 200 µM PA or OA prior to the application of 2 g/cm^2^ compressive force. To further determine the additive impact of bacterial infection, HPdLF were stimulated with lipopolysaccharides (LPS) obtained from *Porphyromonas gingivalis*. In mechanically compressed HPdLF, PA enhanced *COX2* expression and PGE2 secretion. When mechanically stressed HPdLF were additionally stimulated with LPS, the PGE2 and IL6 secretion, as well as monocyte adhesion, were further increased in PA-treated cultures. Our data emphasize that a hyperlipidemic condition enhances the susceptibility of HPdLF to an excessive inflammatory response to compressive forces, when cells are concomitantly exposed to bacterial components.

## 1. Introduction

Obesity is a common, non-communicable disease, and it is well established that adipose tissue is highly metabolically active [1]. Adipocytes and macrophages, the main cell types of adipose tissue, modulate the secretion of several bioactive molecules [2]. This includes pro-inflammatory cytokines and other inflammatory markers, as well as hormone-like signaling mediators, that have therefore been termed adipokines [2]. They have several functions in different organs and contribute to the regulation of lipid metabolism and inflammatory processes [3]. In obese patients, increased release of pro-inflammatory cytokines such as tumor necrosis factor alpha (TNFα), interleukin 6 (IL6), and IL8, as well as IL1β contributes to the dysregulation of biologically relevant processes and promotes low-grade systemic inflammation [4,5,6].

Studies on the underlying mechanisms of obesity-associated inflammation have focused on alterations in lipid metabolism and the resulting elevated serum levels of free fatty acids (FFA), such as saturated palmitic acid (PA) and monounsaturated oleic acid (OA) [4,7,8,9,10,11,12,13,14]. Although they are relevant for normal cell functions [15], hyperlipidemic conditions of both fatty acids impact inflammatory processes in several cell types [16,17,18,19,20,21,22,23].

Saturated fatty acids (SFA) such as PA have been shown to activate pro-inflammatory genes (*TNFα*, *IL6*, *IL8*, *IL1α*, *IL1β*) via multiple pathways [16,17,18,19,20,21,22,24,25]. In a hypothalamic cell line, palmitic acid has been described as an agonist for toll-like receptors (TLR), activating several protein kinases, causing ER stress and increased ROS production, and thus promoting cytokine production and secretion [26]. In addition, culturing cells with palmitic acid was reported to result in the accumulation of palmitoyl-CoA, which serves as a substrate for ceramide production [27]. A high ceramide level is a potent trigger of cell cycle arrest and apoptosis [28], which supports our recent findings of higher cellular senescence and cell death in HPdLF associated with PA [29].

Monosaturated fatty acids (MUFA), such as OA, have mostly been reported to reduce levels of pro-inflammatory cytokines, such as TNFα and IL6, in part by balancing SFA-induced inflammation [8,19,23,30]. However, in dermal fibroblasts, OA appears to be pro-inflammatory, causing increased cyclooxygenase 2 (COX2) expression, higher ROS levels, and oxidative damage [31]. In addition, oleic acid is suspected to predispose obesity and obesity-related disorders by promoting adipogenesis of fibroblast-like 3T3-L1 cells [32]. In vivo, the effects of hyperlipidemia are based on an excess of specific fatty acids and their relation to each other [4,7,8,9,10,11,12,13,14], which complicates in vitro studies.

Similarly to obesity, periodontal inflammation is a global health problem, and patients suffering from severe periodontitis also show low-grade systemic inflammation, with increased levels of pro-inflammatory cytokines [33]. Periodontitis is characterized by the destruction of tooth-supporting soft tissue and alveolar bone through a bacterial-induced inflammatory host response [34]. As the second largest microbial ecosystem, the microbiome of the oral cavity is composed of a variety of different microorganisms including bacteria, fungi, viruses, archaea, and protozoa [35]. *Porphyromonas gingivalis* (*P. gingivalis*) has been classified as one of the major gram-negative oral anaerobes affecting periodontal health [36]. A variety of virulence factors, such as lipopolysaccharides (LPS), gingipains, and fimbriae are responsible for the pathogenic mechanism of *P. gingivalis* [37]. To enable in vitro studies, *P. gingivalis* or its LPS is often used to mimic periodontitis-causing stimuli. It should be noted, however, that none of these factors or *P. gingivalis* alone can trigger periodontitis, but that an interplay of a variety of microbial imbalances and a corresponding host susceptibility is necessary for this to occur. In obese mice, a delay in response to infection with *P. gingivalis* was reported [38]. In addition, *P. gingivalis* LPS was shown to promote the pro-inflammatory profile of adipokines, possibly contributing to obesity-related inflammation [39,40]. Moreover, changes in gut microbiome due to *P. gingivalis* swallowing were reported to induce systemic inflammation and metabolic alterations in animal studies [41,42]. However, whether both diseases can subsequently impact orthodontic tooth movement (OTM) is currently poorly understood.

For successful orthodontic treatment, a controlled and non-excessive inflammatory response of the periodontal tissue is necessary in order to foster relevant processes related to remodeling of the alveolar bone [43]. The RANKL/OPG system is involved in alveolar bone remodeling by mechanical forces, with an increase in inflammatory cytokines promoting osteoclast-activating expression and secretion of receptor activator of nuclear factor kappa-β ligand (RANKL), whereas the counteracting osteoprotegerin (OPG) is significantly reduced [44]. The complex inflammatory signaling cascades due to orthodontic treatments are mainly regulated by periodontal ligament fibroblasts (PdLF), which are the main cell type in the periodontium and located between the teeth and alveolar bone [45]. Dysregulation can result in tooth root degradation or even tooth loss, which are major risks of OTM [46,47]. When teeth are mechanically stressed, the triggered aseptic transient inflammation is modulated area-specifically by PdLF. In particular, the expression and secretion of pro-inflammatory cytokines, such as IL6, IL8, prostaglandin E2 (PGE2), and TNFα, are characteristics of the compression side of the PdL, while the release of anti-inflammatory cytokines, such as IL10, is more prominent on the tensile side [43,48]. This area-specific inflammation of the PdL is important for tissue and bone remodeling, by promoting the degradation and reorganization of the extracellular matrix and vascular supply, as well as the activation and differentiation of osteoblasts and osteoclasts [43,49]. Besides that, PdLF also recognize pathogens and their virulence factors through pattern recognition receptors [45], which also induce an inflammatory cellular response.

To date, only a few studies have investigated whether obesity affects tooth movement [50,51,52,53], and none of the studies additionally addressed effects due to periodontitis. Under obese conditions, faster tooth movement was reported in children and adolescents at early stages of tooth movement one week after force application [51]. Although obese patients were mostly reported to have higher levels of osteoclastic activity, due to pro-inflammatory promotion of RANKL levels [54], OTM resulted in prolonged overall treatment duration [50,53] and reduced the numbers of osteoclasts in force-treated obese mice [52]. However, Bremen et al. mainly based the result of their studies on the poorer cooperation of obese patients during orthodontic therapy. The extent to which the force-induced inflammatory response of PdL is impaired under obese conditions has not been investigated so far.

When tooth movement was simulated in rats suffering from periodontitis, an up-regulated expression of several cytokines in the PdL, as well as an increased number of activated osteoclasts and an enhanced extent of dorsal root resorption, was shown [55]. In contrast, in a mouse-model, induction of periodontal inflammation reduced tooth movement by inhibiting osteoclastogenesis [56], whereas oral administration of *P. gingivalis* in obese mice led to increased alveolar bone loss compared to lean controls [38]. However, to our knowledge, it has not yet been investigated whether periodontitis has an influence on obesity-associated changes in orthodontic force-induced modulation of inflammatory tissue response.

In this context, our aim was: (1) to investigate, whether a fatty acid-simulated hyperlipidemic condition impacts the function of HPdLF in modulating the inflammatory response to a compressive stimulus; and (2) to address changes due to the administration of *P. gingivalis* LPS. In view of an ever-increasing proportion of obese patients suffering from periodontitis, who nevertheless desire orthodontic treatment, this study should provide initial information on the biological background.

## 2. Results

### 2.1. Palmitic Acid Induces an Increased Inflammatory State in HPdLF

**Figure 1:**
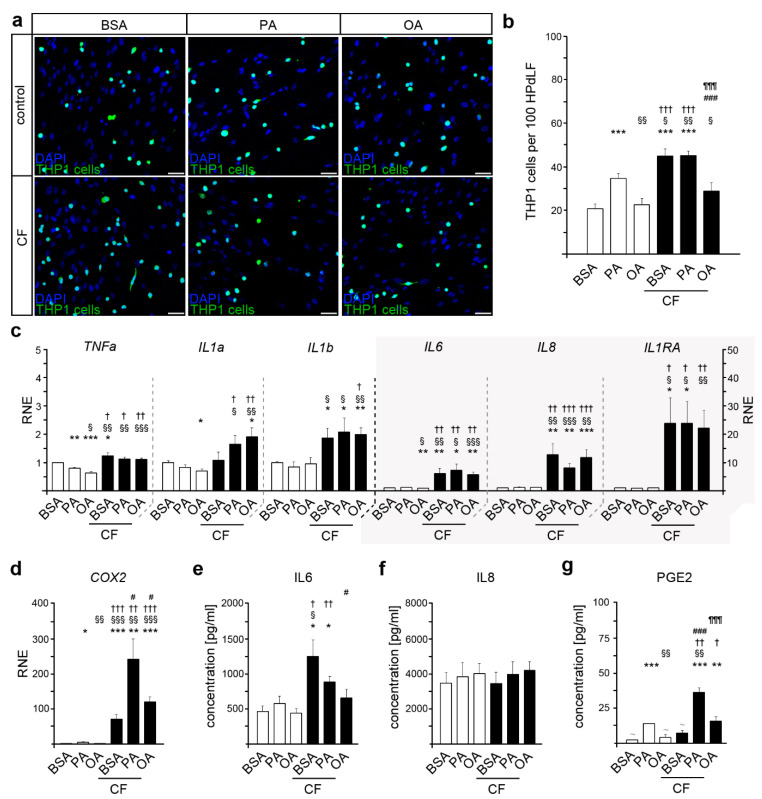
Palmitic and oleic acid influence the inflammatory response of human periodontal ligament fibroblasts (HPdLF) to compressive force of six hours (CF). (**a**,**b**) Analysis of the number of adherent THP1 monocytic cells (green) on HPdLF (blue), stimulated either with palmitic or oleic acid in comparison to BSA control (**a**). THP1 cells were stained with CellTracker™ and the nuclei of all cells were stained with DAPI. The relative number of THP1 cells is displayed per 100 HPdLF (**b**). (**c**,**d**) Quantitative expression analysis of genes coding for inflammatory markers *TNFα*, *IL1α*, *IL1β*, *IL6*, *IL8*, *IL1RA* (**c**), and *COX2* (**d**) in fatty acid-cultured HPdLF stimulated with 6 h of compressive force in comparison to BSA controls. Results are normalized to unstimulated BSA controls. (**e**–**g**) Analysis of secreted cytokines IL6 (**e**), IL8 (**f**), and PGE2 (**g**) in HPdLF cultures stimulated with palmitic or oleic acid and six hours of compressive force compared to BSA controls. * *p* < 0.05; ** *p* < 0.01; *** *p* < 0.001 in relation to BSA, § *p* < 0.05; §§ *p* < 0.01; §§§ *p* < 0.001 in relation to PA, † *p* < 0.05; †† *p* < 0.01; ††† *p* < 0.001 in relation to OA, # *p* < 0.05; ### *p* < 0.001 in relation to BSA+CF, ¶¶¶ *p* < 0.001 in relation to PA + CF; one-way ANOVA and post hoc test (Tukey). Scale bars: 50 μm in (**a**). BSA, bovine serum albumin; CF, compressive force; OA, oleic acid; PA, palmitic acid; RNE, relative normalized expression; ^~^, below detection limit.

To investigate the impact of hyperlipidemic palmitic and oleic acid levels on the regulatory function of HPdLF, we performed a THP1 cell adhesion assay (Figure 1a,b). Non-adherent THP1 monocyte-like cells are attracted by the cytokines secreted by stimulated HPdLF and can differentiate into functional adherent macrophages [57]. We analyzed the number of adherent Alexa488-labeled THP1 cells on HPdLF cultured in palmitic or oleic acid. Cells incubated with BSA only were used as a control, since BSA functions as a carrier for fatty acids. We detected an increased number of THP1 cells on PA-incubated HPdLF (Figure 1a,b). Cultivation with OA resulted in similar amounts of attracted THP1 monocytic cells compared to the BSA controls. This suggests that palmitic acid in particular promotes an inflammatory response in HPdLF.

### 2.2. Oleic Acid Impacts THP1 Adhesion due to Mechanical Compression in HPdLF

The compressive forces associated with orthodontic procedures promote inflammatory processes that are modulated by periodontal ligament cells [43,45]. Therefore, we investigated whether culturing with fatty acids affects the response of HPdLF to six hours of mechanical compression, at which time the expression and secretion of several cytokines are already increased [58]. While compression induced increased THP1 cell adhesion in the BSA controls and PA-treated HPdLF (Figure 1a,b), OA supplementation hindered the force-induced increase in THP1 attraction. Thus, our data suggest that oleic acid limits the inflammatory response to a compressive stimuli of six hours.

### 2.3. COX2 Expression and PGE2 Secretion Are Altered in Mechanically Stimulated HPdLF in Relation to Fatty Acid Stimulation

To further analyze the inflammatory response of HPdLF cultured in fatty acids, we performed quantitative PCR of genes coding for the cytokines and inflammatory markers, *TNFα*, *IL1α*, *IL1β*, *IL1RA*, *IL6*, *IL8,* and *COX2* (Figure 1c,d). We detected no changes in the baseline levels of *IL1β*, *IL1RA,* and *IL8*, whereas *TNFα* was reduced under both fatty acid conditions (Figure 1c). Moreover, OA cultures showed significantly lower levels of *IL1α* and *IL6* (Figure 1c). In PA-treated HPdLF, we detected increased expression of *COX2* compared to BSA controls (Figure 1d).

As a result of the six-hour compressive stimuli, the expression of most genes was increased regardless of the culture condition (Figure 1c,d). However, the force-induced increase in *COX2* expression was significantly higher in PA and OA cultures compared to BSA controls (Figure 1d). This increase was still significantly lower in OA-treated HPdLF than in the respective PA cultures.

Analysis of cytokine secretion in the supernatant revealed no significant changes of basic IL6 levels with fatty acid treatment. However, mechanical stimulation promoted IL6 secretion in the BSA controls, as well as in PA cultures, but not in OA-treated HPdLF (Figure 1e). In contrast, IL8 secretion was not altered, neither by fatty acid treatment nor mechanical compression (Figure 1f).

In contrast to the BSA controls, whose PGE2 levels were below the detection limit, we detected PGE2 secretion by HPdLF treated with PA (Figure 1g). In addition, mechanical compression increased the PGE2 levels in PA cultures and raised PGE2 levels slightly above the detection limit in OA cultures.

Since IL6, rather than PGE2, is highly important for monocytes for differentiating into macrophages [59,60], our analysis of inflammatory markers supported the previous results of the THP1 assay.

### 2.4. Stimulation with Lipopolysaccharides from P. gingivalis Resulted in an Excessive Inflammatory Response of HPdLF Exposed to Palmitic Acid

**Figure 2:**
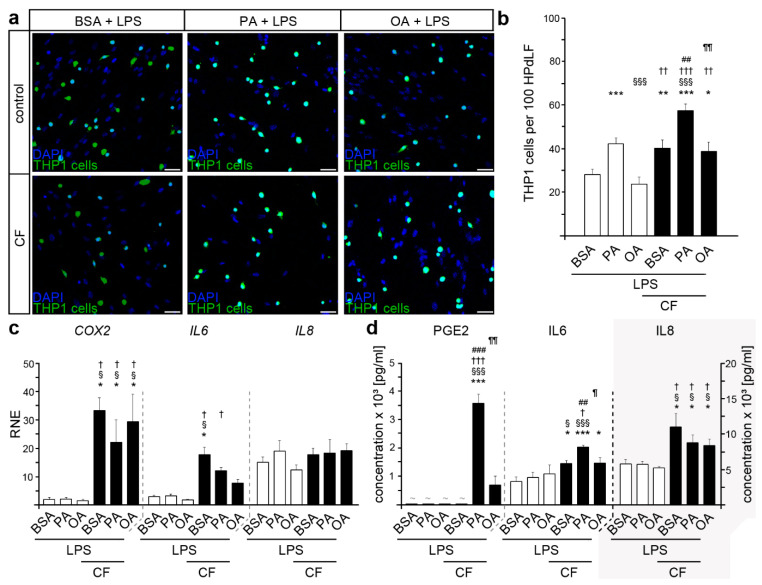
Stimulation with LPS obtained from *P. gingivalis* led to an excessive immune response in palmitate-cultured HPdLFs undergoing compressive stress. (**a**,**b**) Analysis of the number of adherent THP1 monocytic cells (green) on HPdLF (blue) stimulated either with palmitic or oleic acid in comparison to BSA controls and stimulated for six hours with compressive force (CF) (**a**). THP1 cells were stained with CellTracker™ and the nuclei of all cells were stained with DAPI. The relative number of THP1 cells is displayed per 100 HPdLF (**b**). (**c**) Quantitative expression analysis of genes coding for inflammatory markers *IL6*, *IL8,* and *COX2* in fatty acid-cultured HPdLF stimulated with 6 h compressive force in comparison to BSA controls. Results are normalized to unstimulated BSA controls. (**d**) Analysis of secreted cytokines PGE2, IL6, and IL8 in HPdLF stimulated with palmitic or oleic acid and 6 h of compressive force compared to BSA controls. * *p* < 0.05; ** *p* < 0.01; *** *p* < 0.001 in relation to BSA, § *p* < 0.05; §§§ *p* < 0.001 in relation to PA, † *p* < 0.05; †† *p* < 0.01; ††† *p* < 0.001 in relation to OA, ## *p* < 0.01; ### *p* < 0.001 in relation to BSA+CF, ¶ *p* < 0.05; ¶¶ *p* < 0.01 in relation to PA + CF; one-way ANOVA and post-hoc test (Tukey). Scale bars: 50 μm in (**a**). BSA, bovine serum albumin; CF, compressive force; LPS, lipopolysaccharides of *P. gingivalis*; OA, oleic acid; PA, palmitic acid; RNE, relative normalized expression; ~, below detection limit.

In order to simulate an infection with *P. gingivalis*, HPdLF were stimulated for 24 h with the appropriate lipopolysaccharides. In comparison to the unstimulated BSA controls, LPS stimulation resulted in an increased THP1 cell adhesion (compare with Figure 1b, Figure 2a,b; *p*-value = 0.03473, *). This was also detectable in the LPS-stimulated PA cultures (*p*-value = 0.04951, *). Additionally, higher numbers of adherent THP1 cells were evident in LPS-stimulated PA cultures compared to the respective BSA controls (Figure 2a,b).

After applying a compressive stimuli, a significant increase in THP1 cell adhesion was detected in all LPS-stimulated HPdLF (Figure 2a,b). However, the force-induced increase in adherent THP1 cells was significantly higher in LPS-primed PA cultures. In addition, the LPS stimulation of compressed PA-cultures led to significantly higher numbers of adherent TPH1 cells, when compared to those not stimulated with LPS (compare with Figure 1b; BSA CF: *p*-value = 0.28094; PA CF: *p*-value = 0.00483, **; OA CF: *p*-value = 0.11800).

A quantitative analysis of *COX2* expression showed no fatty acid-related differences when stimulated with LPS (Figure 2c). Nevertheless, the transcriptional levels were significantly higher compared to those of non-LPS-stimulated HPdLF (compare with Figure 1d; BSA *p*-value = 0.03516, *; PA: *p*-value = 0.01895, *; OA: *p*-value = 0.01001, *). Similar changes in expression were also found for *IL6* and *IL8* (Figure 2c). Compared to their respective unstimulated conditions (compare with Figure 1c), the application of LPS led to a significantly up-regulated expression in BSA controls (*IL6 p*-value = 0.00310, **; *IL8 p*-value = 4.29214 × 10^−8^, ***), in PA cultures (*IL6 p* value = 0.00368; **; *IL8 p* value = 6.02460 × 10^−5^; ***), and in OA-cultured HPdLF (*IL6 p*-value = 0.02475, *; *IL8 p*-value = 2.21541 × 10^−6^; ***). In response to compressive forces, increased values were detected for *COX2* and *IL6* transcription, but not for *IL8* (Figure 2c). Moreover, *IL6* levels showed significant differences regarding fatty acid stimulation, with reduced expression in OA cultures.

Further analysis of the secreted proteins in the culture media revealed a profound increase of PGE2 in the mechanically stressed and LPS-stimulated PA cultures (Figure 2d) that was significantly higher compared to the non-LPS-stimulated PA cultures (compare with Figure 1g; *p*-value = 0.00042, ***). Under all other conditions, PGE2 levels were below the detection limit. In addition, we detected a force-induced enhancement of IL6 but not of IL8 secretion levels for LPS-stimulated BSA controls (Figure 2d). The increase in IL6 cytokine release was even higher when cells were additionally exposed to PA, which further supports the modulatory impact of PA on the inflammatory response of HPdLF to mechanical, as well as bacterial, stress. Moreover, we detected significantly higher levels of IL6 and IL8 secretion in LPS-stimulated PA and OA cultures compared to the HPdLF that were not challenged by *P. gingivalis* LPS (compare with Figure 1e,f; IL6 PA *p*-value = 3.18950 × 10^−5^, ***; OA *p*-value = 0.02287, *; IL8 PA *p*-value = 0.00142, **; OA *p*-value = 0.00103, **). For IL8, this was also detected for BSA controls (*p*-value = 2.06347 × 10^−5^, ***).

In summary, our data point to an increased inflammatory response in mechanical HPdLF treated with palmitic acid. Furthermore, our data strongly suggest that the inflammatory stress response is even more pronounced when the cells are additionally stimulated with periodontitis-causing bacterial compounds.

## 3. Discussion

In this study we investigated the possible impact of a fatty acid-stimulated hyperlipidemic condition, typically seen in obese patients, on the function of human periodontal ligament fibroblast, in terms of their inflammatory response to compressive forces when additionally challenged with *P. gingivalis* LPS. Exposition to palmitate resulted in enhance inflammatory state, even when not additionally stressed. Mechanical forces increased *COX2*/PGE2 levels in fatty-acid cultures and additional LPS administration further increased PGE2 and IL6 secretion in PA-primed HPdLF.

The causes and mechanisms of obesity-induced inflammatory processes are not fully understood. However, the important role of fatty acids in the activation and modulation of inflammatory signaling pathways is suggested. The applied concentration of both fatty acids, PA and OA, has previously been used in other in vitro studies, and the used BSA concentration relates to serum albumin and is characteristic for obesity and hypertriglyceridemia [9,10,11,12,13,14,29]. The investigated cytokines play important roles in the defense against pathogens in periodontal diseases, as well as in the regulation of orthodontic force-induced alveolar bone remodeling [43,61]. Dysregulation by hyperlipidemic conditions could affect inflammation, which is important for both processes, and a basic investigation of the underlying biological changes of inflammatory markers would be relevant for further studies in obese patients with periodontal inflammation.

We detected increased *COX2* expression and PGE2 secretion in HPdLF that were stimulated with palmitic acid. This is in line with recent studies in which PA-related increased *COX2* transcription levels were reported in several cells types [20,62,63]. Catalyzing a rate-limiting step in prostaglandin synthesis, COX2 overexpression could lead to increased production of PGE2, which was also shown to induce fibroblast apoptosis by multiple pathways [64]. Moreover, intracrine COX2/PGE2 signaling was also reported to contribute to the establishment and maintenance of cellular senescence [65]. Therefore, our recently reported higher numbers of apoptotic and senescent HPdLF cultured in palmitate may have been due to, at least partially, increases in *COX2*/PGE2 signaling [29]. In contrast to our results, de Souza et al. [20] also showed enhanced expression of *IL6* in response to PA treatment in human endothelial cells. However, the cell cultures were simultaneously stimulated with TNFα, which could have influenced the expression pattern of several cytokines. In orbital fibroblasts, comparable concentrations of palmitate promoted the secretion of IL6 and *monocyte chemotactic protein 1* (MCP1) [66]. It should be noted that the preparation of the corresponding palmitate concentration was done with ethanol, rather than heated water, and thus could potentially have impacted the cytokine levels. Higher concentrations of PA also resulted in increased levels of the pro-inflammatory cytokines IL6 and TNFα in cardiac fibroblasts [16]. However, these high concentrations were shown to be toxic in HPdLF (data not shown). In cultured HPdLF, reduced expression levels of *TNFα*, *IL1α,* and *IL6* were detected due to oleate treatment. Comparable observations were also made in other cell types stimulated with OA alone [22,23], which underline the anti-inflammatory potential of oleate. However, in murine dermal fibroblasts, OA induced pro-inflammatory cellular responses via increased COX2 levels [31]. Therefore, it can be assumed that fatty acids evoke a specific cellular reaction depending on cell type and their ratio to other fatty acids, as well as additional intrinsic and extrinsic stimuli. However, for simulating an obesity-related pro-inflammatory state, hyperlipidemic culturing with PA seems to be favorable for HPdLF.

During OTM, the compressive force acts as a strong extrinsic stimulus to HPdLF. Here, we could show that hyperlipidemic fatty acid levels influence the force-induced increase in *COX2* expression in mechanically stimulated HPdLF. Accordingly, the PGE2 secretion of compressed HPdLF was also increased in response to different fatty acids. In general, the PGE2 secretion of HPdLF is promoted by mechanical stimulation in vitro and in vivo and it is responsible, among other cytokines, for the initiation of osteoclastic activity [67,68]. Some studies reported a rather moderate increase in PGE2 after OA supplementation [69,70], which is also supported by our findings.

Too high levels of PGE2 could be problematic for tissue homeostasis and bone remodeling. In animal model systems, submucosal as well as intraligamentous PGE2 administration significantly accelerated tooth movement with increased root resorption [67,71,72]. Moreover, high PGE2 levels were also associated with enhanced transformation of monocytes into osteoclasts and the inhibition of PdL fibroblast proliferation [73]. Contrary to these results, other studies showed that elevated PGE2 levels inhibited osteoclast formation and function [74,75]. We recently hypothesized that mechanically compressed HPdLF that were exposed to palmitic acid would fail to sufficiently activate immature osteoclasts, due to enhanced cellular senescence and cell death [29]. Our data now suggest that a high level of PGE2 secretion by compressed PA-stimulated HPdLF could directly inhibit the differentiation of immature osteoclasts. This would support the results of Yan et al. [52], who demonstrated reduced numbers of osteoclasts in obese mice, correlating with attenuated experimental tooth movement. Further studies on patient PGE2 levels could prove whether this theory is relevant to the increased duration of orthodontic treatment in obese adolescents [50]. However, it should be taken into account that higher levels of several cytokines are also present in senescent cells, as well as in cell death [76,77]. Although cell survival is generally not affected by compressive stimulation with 2g/cm² [78], fatty acid primed HPdLF might be more sensitive to the applied forces.

In contrast to several studies that showed enhanced IL6 levels due to palmitic acid exposition [24,79,80], we could not detect increased levels of either IL6 expression or secretion in PA-treated HPdLF. In nondiabetic peripheral blood mononuclear cells, palmitic acid also failed to increase IL6 secretion [81]. However, a force-induced increase in IL6 secretion was observed in BSA controls and evident in PA-treated compressed HPdLF. Interestingly, compressed HPdLF exposed to OA did not show increased IL6 levels. Our data support the findings of Rodrigues et al. [82], who detected reduced IL6 concentrations in the skin wounds of rats fed with higher amounts of oleic acid. As an important osteotropic cytokine, force-released IL6 directly or indirectly interacts with bone-modulating cells, thereby promoting bone resorption on the compressive side of affected teeth [43]. As a result, reduced IL6 levels, as found in OA-treated HPdLF cultures, could lead to decreased bone resorption, which would hamper tooth movement and probably increase treatment duration. It should be taken into account that hyperlipidemia in obese patients is not represented only by the increase of a particular fatty acid, but is based on a composition of several SFA, MUFA, and polyunsaturated fatty acids (PUFA). In the context of our study, we can only speculate how obesity-related hyperlipidemia might affect osteoclastogenesis. However, in vitro preliminary studies can help in the targeted analysis of limited patient material.

Since a controlled inflammation contributes to the regulation of force-induced alveolar bone remodeling, several studies have focused on the impact of periodontal disease, an uncontrollable inflammatory stimulus when left untreated during OTM. *P. gingivalis*, a major periodontal pathogen, induces an inflammatory response in HPdLF via increased expression and secretion of IL6 and IL8, respectively [83,84]. We detected elevated levels of these cytokines in LPS-stimulated HPdLF, which, interestingly, were independent of the presence of fatty acids. For IL8, this appears to be consistent with the study of Fadel et al. [85], who detected no differences in IL8 levels between obese and normal weight patients suffering from periodontitis. In the case of IL6, however, this seems to be different from other studies reporting that *P. gingivalis* augmented palmitate-induced cytokine secretion in gingival fibroblasts and osteoclasts [86,87]. In these studies, different cell types were investigated and a lower palmitate concentration [86] or direct bacterial infection [87] was used for stimulation.

Based on our data, we speculate that palmitic acid in combination with *P. gingivalis* LPS stimulation makes HPdLF susceptible to an excessive immune response to compressive forces. This is based on the detection of excessive PGE2 secretion, as well as increased IL6 amounts and higher levels of adherent THP1 monocytic cells on palmitate-treated HPdLF that were LPS and force-stimulated. In this context, Sokolova et al. [16] reported that cardiac fibroblast exposed to palmitate and additionally challenged with LPS from *Escherichia coli* (*E. coli*) showed an increased expression and secretion of the pro-inflammatory cytokine IL1β, which was fatty acid dose- and time-dependent. In the same study, they revealed that stimulation with oleic acid and *E. coli* LPS did not induce comparable changes in IL1β levels.

The results of our study are limited by their experimental design. First, we examined only a certain amount of PA and OA as well as *P. gingivalis* LPS, and second, the compressive force was applied only for the specific duration of six hours. Thus, we cannot exclude the possibility that changes in the experimental setup could cause further changes in the expression and secretion of the cytokines studied, which are not obvious under our experimental design. However, this requires further investigation. Furthermore, it is important to note that periodontitis is not caused by overexposure to the LPS of one bacterium, but by a variety of virulence factors of different pathogens, as well as the specific host immune system. However, our studies provide some first ideas for the possible biological mechanisms of how obesity-related hyperlipidemia and periodontitis concomitantly affect orthodontic tooth movement, which can be further addressed in future studies.

## 4. Materials and Methods

### 4.1. Cell Culture

Commercially acquired human periodontal ligament fibroblast (HPdLF, Lonza, Basel, Switzerland) were grown in culture medium consisting of Dulbecco’s modified Eagle medium (DMEM; Thermo Fisher Scientific, Carlsbad, CA, USA) containing 4.5 g/L glucose, 10% heat-inactivated fetal bovine serum (Thermo Fisher Scientific, Carlsbad, CA, USA), 100 U/mL penicillin, 100 µg/mL streptomycin, and 50 µg/mL L-ascorbic acid at 37 °C, 5% CO_2_ and 95% humidity. Cells were passaged when reaching a confluency of 75% with 0.05% Trypsin/EDTA (Thermo Fisher Scientific, Carlsbad, CA, USA). For experiments, HPdLF of passage four to eight were used.

THP1 monocytic cells (DMSZ, Braunschweig, Germany) were cultured in RPMI 1640 medium (Thermo Fisher Scientific, Carlsbad, CA, USA) containing 10% FBS, 100 U/mL penicillin, and 100 µg/mL streptomycin at 37 °C, 5% CO_2_, and 95% humidity. The non-adherent cells were passaged regularly after seven days and seeded at a density of 1 × 10^6^ cells in 20 mL medium in T175 culture flask (Thermo Fisher Scientific, Carlsbad, CA, USA). For this, cells were pelleted by centrifugation for 5 min at 1000× *g* and diluted in 1 mL RPMI culture medium prior to cell counting in a hemocytometer (Neubauer Chamber Improved, Avantor, Radnor, PA, USA).

### 4.2. Fatty Acid and P. gingivalis LPS Stimulation

For the analysis of RNA expression and cytokine secretion, 2.5 × 10^4^ HPdLF were seeded into each well of a 6-well plate. For TPH1 cell adherence assay, 5 × 10³ cells were plated onto coverslips into each well of a 24-well-plate. Prior to fatty acid stimulation, cells were cultured in DMEM culture medium for 24 h. The stimulation with 200 µM palmitic or 200 µM oleic acid was performed as described previously [29]. Briefly, fatty acids were dissolved at 70 °C in sterile water containing 50 mM NaOH, complexed with 37 °C preheated bovine serum albumin (BSA, Seqens IVD, Limoges, France) and diluted in culture medium. As control, 0.66% BSA in DMEM culture medium was used. Fatty acid treatment was performed for six days resulting in a cell confluence of 65–75% before a compressive force was applied. For bacterial stimulation, 10 µg/mL lipopolysaccharides of *P. gingivalis* (InvivoGen, San Diego, CA, USA) were added to the culture medium 24 h before mechanical stimulation. To control for LPS application, HPdLF were stimulated with LPS but not mechanically loaded.

### 4.3. Mechanical Compression

Application of compressive force in 6-well plates was performed on the basis of the protocol of Kirschneck et al. [88] and as previously described [29]. Briefly, a compressive stimuli of 2 g/cm^2^ was applied with sterile glass plates for six hours at 37 °C, 5% CO_2_, and 95% humidity. Then, cells were either directly isolated with TRIzol Reagent (Thermo Fisher Scientific, Carlsbad, CA, USA) for expression analysis or medium was collected 24 h later for protein analysis.

The application of compressive forces in 24-well plates was performed by centrifugation for six hours at 30 °C. A force of 7.13 g/cm² was applied, as this was the minimal conditions of the centrifuge. Control cells were cultured at 30 °C for the time of the mechanical stimulation.

### 4.4. THP1 Cell Adherence assay

To visualize the inflammatory response of HPdLF to fatty acid stimulation, as well as to mechanical and bacterial stress, a THP1 cell adhesion assay was performed. For this, non-adherent THP1 monocytic cells were first stained with 15 µM Celltracker CMFDA (Thermo Fisher Scientific, Carlsbad, CA, USA) in sterile phosphate buffered saline (PBS, Thermo Fisher Scientific, Carlsbad, CA, USA) for 30 min at 37 °C, 5% CO_2_, and 95% humidity. Cells were then pelleted by centrifugation (5 min, 1000× *g*), resuspended in RPMI medium, and 50 × 10³ cells were added to each well of cultured HPdLF. Cell adhesion was carried out for 30 min before non-adhered THP1 cells were removed by two washing steps with prewarmed sterile PBS. After treatment, cells were fixated in 4% paraformaldehyde for 10 min, washed in PBS, and nuclei were stained for 5 min with DAPI (1:10,000 in PBS). Coverslips were embedded with Mowiol^®^ 4–88 (Carl Roth, Karlsruhe, Germany) on glass object slides for microscopic imaging. The experiment was repeated three times, with two coverslips per condition.

### 4.5. RNA Extraction and Quantitative PCR

For expression analysis, the RNA of treated HPdLF was isolated with TRIzol Reagent (Thermo Fisher Scientific, Carlsbad, CA, USA)/1-bromo-3-chloropropane and purified with an RNA Clean and Concentrator-5 kit (Zymo Research, Freiburg, Germany) according to the manufacture’s guidelines. RNA quantity and quality was tested with Nanodrop 2000 (Avantor, Radnor, PA, USA). SuperScript IV Reverse Transcriptase (Thermo Fisher Scientific, Carlsbad, CA, USA) was used for cDNA synthesis using Oligo(dt)_18_ primers (Thermo Fisher Scientific, Carlsbad, CA, USA), according to the manufacture’s protocol. Quantitative PCR was performed with Luminaris Color HiGreen qPCR Master Mix (Thermo Fisher Scientific, Carlsbad, CA, USA), according to the manufacture’s protocol and analyzed with qTOWER3 (Analytik Jena, Jena, Germany). Primer sequences for all analyzed genes are displayed in Table 1. *RPL22* and *TBP* were used as reference genes. Melting curve analysis and agarose gel electrophoresis was performed to assess primer quality and specificity. A dilution series was used to calculate primer efficiency. Data were analyzed with the efficiency corrected ΔΔCT method [89]. Each condition was analyzed at least in biological triplicate, with technical duplicates per sample.

### 4.6. Enzyme-Linked Immunosorbent Assay (ELISA)

To analyze cytokine secretion, IL6 (R&D Systems, Minneapolis, MN, USA), IL8 (R&D Systems, Minneapolis, MN, USA), and prostaglandin E2 (PGE2; Thermo Fisher Scientific, Carlsbad, CA, USA) ELISA were performed on medium isolated from HPdLF, according to the manufacturer’s guidelines. Each individual condition was tested at least in biological triplicate, with technical duplicates per sample.

### 4.7. Microscopy, Image Analysis, and Statistics

The THP1 cell adhesion assay was imaged with an inverted confocal laser scanning microscope TCS SP5 (Leica), and *Fiji* software (https://imagej.net/Fiji, accessed on 1 April 2017) was used for cell number analysis. *Graph Pad Prism* (https://www.graphpad.com, accessed on 1 February 2021) was used for statistical analysis, in addition to *Adobe Photoshop CS5* (https://adobe.com, accessed on 1 February 2013) for figure illustration. One-way ANOVA and post hoc test (Tukey) were used as statistical tests. Significance levels: *p* value < 0.05 *; *p* value < 0.01 **; *p* value < 0.001 ***.

## 5. Conclusions

Our study provides new information on how obesity-related hyperlipidemia affects the function of periodontal ligament fibroblasts in modulating the inflammatory response to compressive forces in vitro. Force-induced inflammation is enhanced by palmitate and further increased when cells were additionally challenged with LPS from *P. gingivalis*. Thus, this study provides the first information on changes in the regulation of cytokines that may be relevant during orthodontic tooth movement in an ever-increasing proportion of obese patients who also suffer from periodontitis.

## Figures and Tables

**Table 1:** qPCR primer sequences of human genes indicated in 5’-3’ direction. bp, base pairs. Length, amplicon length.

Gene	Gene Symbol	NCBI Gene ID	Primer Sequence	Length
C-X-C motif chemokine ligand 8	*IL8*	3576	fw TTGGCAGCCTTCCTGATTTCTrew GGTCCACTCTCAATCACTCTCA	149 bp
Interleukin 1 alpha	*IL1α*	3552	fw GACTGCCCAAGATGAAGACCArev CCAAGCACACCCAGTAGTCT	185 bp
Interleukin 1 beta	*IL1β*	3553	fw CGAATCTCCGACCACCACTArev AGCCTCGTTATCCCATGTGT	186 bp
Interleukin-1 receptor antagonist	*IL1RN* *(IL1RA)*	3557	fw GATGTGCCTGTCCTGTGTCArev ACTCAAAACTGGTGGTGGGG	146 bp
Interleukin 6	*IL6*	3569	fw CATCCTCGACGGCATCTCAGrew TCACCAGGCAAGTCTCCTCA	164 bp
Prostaglandin-endoperoxide synthase 2	*PTGS2* *(COX2)*	5743	fw GATGATTGCCCGACTCCCTTrew GGCCCTCGCTTATGATCTGT	185 bp
Ribosomal protein L22	*RPL22*	6146	fw TGATTGCACCCACCCTGTAGrev GGTTCCCAGCTTTTCCGTTC	98 bp
TATA-box binding protein	*TBP*	6908	fw CGGCTGTTTAACTTCGCTTCCrev TGGGTTATCTTCACACGCCAAG	86 bp
Tumor necrosis factor	*TNFα*	7124	fw CACGCTCTTCTGCCTGCTGrev AGGCTTGTCACTCGGGGTT	130 bp

## Data Availability

The datasets of this study are available from the corresponding author on reasonable request. The data are not publicly available due to very large size of microscopy images.
